# Cancer Microenvironment and Endoplasmic Reticulum Stress Response

**DOI:** 10.1155/2015/417281

**Published:** 2015-09-27

**Authors:** Claudia Giampietri, Simonetta Petrungaro, Silvia Conti, Antonio Facchiano, Antonio Filippini, Elio Ziparo

**Affiliations:** ^1^Istituto Pasteur-Fondazione Cenci Bolognetti, Department of Anatomy, Histology, Forensic Medicine and Orthopedics, Section of Histology and Medical Embryology, Sapienza University of Rome, 00161 Rome, Italy; ^2^Istituto Dermopatico dell'Immacolata (IDI-IRCCS), 00167 Rome, Italy

## Abstract

Different stressful conditions such as hypoxia, nutrient deprivation, pH changes, or reduced vascularization, potentially able to act as growth-limiting factors for tumor cells, activate the unfolded protein response (UPR). UPR is therefore involved in tumor growth and adaptation to severe environments and is generally cytoprotective in cancer. The present review describes the molecular mechanisms underlying UPR and able to promote survival and proliferation in cancer. The critical role of UPR activation in tumor growth promotion is discussed in detail for a few paradigmatic tumors such as prostate cancer and melanoma.

## 1. Introduction

The cellular environment is constantly changing; thus physiological adaptive responses arise in order to maintain the overall cellular equilibrium and tissue homeostasis. Within such framework, numerous ways have evolved to allow optimal adaptation to environmental stress or, under extreme damage conditions, to remove diseased cells and to prevent toxicity [1].

The endoplasmic reticulum (ER) is the intracellular organelle controlling intracellular Ca^2+^ homeostasis, lipid synthesis, and protein folding. Protein folding occurring in the ER is extremely sensitive to environmental changes regarding redox state, nutrient and Ca^2+^ levels, protein synthesis rate, occurrence of pathogens or inflammatory stimuli, altering protein folding, and ultimately causing accumulation of unfolded or misfolded proteins. This condition is generally known as “ER stress” [2] and a sensitive surveillance mechanism ensures degradation of misfolded proteins [3] preventing entry of misfolded proteins in the secretory pathway. When ER stress occurs, ER functions are altered and a number of molecular actions, collectively named “*unfolded protein response*” (UPR), are activated to counteract the ER stress-associated damages. The UPR has a dual function: it mitigates damage associated with ER stress and, if this is not possible, it activates apoptosis [1]. ER stress response/UPR signaling pathways are activated in primary solid tumors as a result of cell-intrinsic defects, such as dysregulation of protein synthesis, folding, and secretion, and also as a consequence of microenvironment changes. Solid tumors microenvironment differs from normal tissues microenvironment, the former being characterized by nutrient (e.g., glucose) deprivation, low pH, hypoxia, and imbalance between production and removal of reactive oxygen species (i.e., oxidative stress) [4, 5].

All such environmental factors contribute to ER stress and cancer cells select effective ways to adapt and prevent ER stress-induced apoptosis [6, 7].

Recent studies have investigated in detail the different ways utilized by cancer cells, under ER stress conditions, to perturb ER-associated cell death signaling and to promote tumor growth [8, 9]. In the present review the known UPR pathways are summarized; then the different ER stressors acting in cancer microenvironment are reported and ultimately the altered ER stress responses in cancer are described, emphasizing their possible therapeutic implications.

## 2. ER Stress Response

Unfolded protein response (UPR) is a cellular response connecting the ER to the nucleus [10]. It represents a key cellular signaling process investigated since the early nineties in yeast [11, 12].

**Figure 1:**
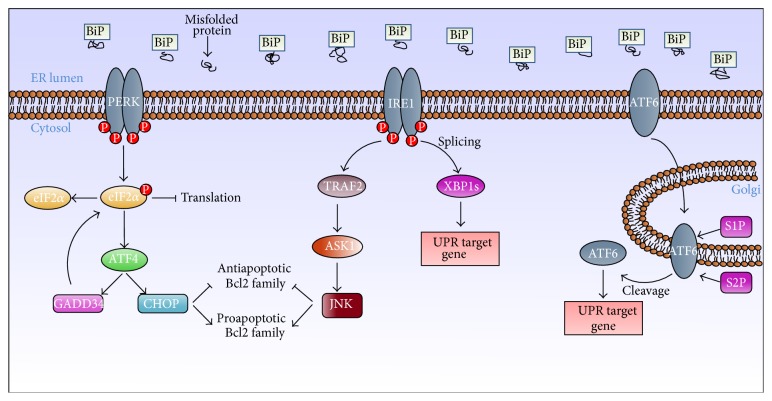
As a consequence of ER stress cells activate signal transduction pathways collectively known as unfolded protein response (UPR). The figure represents the three branches of the UPR and the corresponding UPR sensors (PERK, IRE1, and ATF6).

As shown in Figure 1, three ER-associated proteins are key players of UPR, namely, Pancreatic ER Kinase (*PERK*) [13], Inositol-Requiring Enzyme 1 (*IRE1*) [14], and Activating Transcription Factor 6 (*ATF6*) [15]. Under normal conditions, such three transmembrane proteins are bound and inactivated by a chaperone, Glucose Regulated Protein 78 (BiP, also known as GRP78) [16]. As response to ER stress, BiP dissociates from the UPR sensors to allow their proper signaling [17]. The activation of the ER stress sensors and of their downstream targets halts new proteins transcription and increases the synthesis of molecular chaperones. As a first consequence, the UPR promotes cell survival by enhancing ER ability to fold proteins and preventing further protein accumulation that might exacerbate the ER damage. If such response is not sufficient and the stress persists, the UPR leads to apoptosis [18]. Although the exact molecular mechanisms involved are not known, several evidences suggest that cell death induced by ER stress requires continuous signals exchange between ER and mitochondria [19]. This communication depends on the presence of a physical link between the two organelles, represented by specific contact sites between membranes of the ER and mitochondria, known as Mitochondrial Associated Membranes (MAMs) [20]. The integrity of this interaction is modulated by different proteins, as we recently demonstrated [21], and is essential to maintain cellular homeostasis and to modulate important processes such as apoptosis, ER stress, and autophagy [22].

The three ER stress sensors PERK, IRE1, and ATF6 are characterized by an amino-terminal domain important for the stress perception, maintained in an inactive state by interaction with the chaperone BiP under physiological conditions, and a carboxy-terminal domain that interacts with the transcriptional and translational apparatus [16].

*PERK* activation in response to ER stress leads to phosphorylation of the *α*-subunit of eukaryotic initiation factor 2 (eIF2*α*) which, in turn, blocks protein translation. This event promotes cell survival by preventing further ER damage from other nascent proteins [13]. Thus PERK activation initially leads toward a protective cell survival response; however, stress persistence induces the transcription of C/EBP homologous protein (CHOP), a transcription factor positively controlled by the transcription factor 4 (ATF4). Such event is critical to control the shift from survival to apoptosis. Phosphorylated eIF2*α* activates ATF4, which, in turn, acts on target proapoptotic genes such as growth arrest and DNA damage-inducible 34 (GADD34) and CHOP [23]. CHOP moves to the nucleus, upregulates its proapoptotic target genes, and facilitates the programmed cell death upon ER stress [24].

*IRE1*, activated in response to unfolded proteins accumulation, determines the splicing of a 26-nucleotide-long intron from the mRNA encoding the transcription factor X box-binding protein 1 (XBP1) [25]. The generated splicing variant, XBP1s, acts as a transcription factor that moves to the nucleus and causes the transcription of genes coding ER chaperones, in order to mitigate the stress [26]. IRE1 overexpression has been also shown to trigger apoptosis [27]. IRE1 has been demonstrated to recruit the adapter molecule TNF-receptor-associated factor 2 (TRAF2); the complex TRAF2-IRE1 activates a proapoptotic signal by inducing Apoptosis Signal Regulated Kinase (ASK1), which, in turn, transmits the death signal to c-Jun N-terminal kinase (JNK). Once activated, JNK is responsible for the phosphorylation of Bcl2 thus abolishing its antiapoptotic activity [28]; moreover, it is able to determine the phosphorylation of the proapoptotic proteins BAX and BIM [29, 30], enhancing their proapoptotic effect. According to such complex mechanism, it can be concluded that when the stress persists, PERK and IRE1 signaling cascades can converge, mediating the induction of apoptosis. IRE1 RNase is also involved in a process called RIDD (RNA IRE1-Dependent Decay), consisting in cleavage of mRNAs encoding many different proteins and aimed at maintaining ER homeostasis. It has been found that RIDD activity increases as function of ER stress and correlates with apoptosis induction [31].

In response to ER stress,* ATF6* dissociates from the ER membrane and moves to the Golgi apparatus, where its cytoplasmic domain undergoes a proteolytic cleavage by serine proteases S1P and S2P, resulting in the formation of an active transcription factor [15]. Activated ATF6 goes to the nucleus and promotes the transcription of target genes encoding for different proteins such as BiP, GRP94, protein-disulfide isomerase (PDI), and XBP1 that enhance ER ability to fold accumulated proteins, contributing to restoring initial homeostasis.

## 3. ER Stress Pathways and Cancer

Cancer cells are known to be very resistant to extreme environmental stress and an increasing number of studies indicate that this may be largely due to an altered state of the UPR. The role ER stress and UPR play in cancer is still not completely clarified; however different components are known to be involved and may prove to be promising targets in future anticancer therapy [1].

Cancer cells adaptation to adverse conditions mostly relies on their ability to prevent ER stress-induced apoptosis and perturb the ER stress-associated signaling. A selective advantage occurs in premalignant cells harboring gene mutations able to suppress UPR-induced apoptosis or senescence [2].

Cancer cells have unique modifications enabling them to exploit ER stress responses to promote survival and growth. The ER protein chaperone BiP is commonly found to be highly expressed in breast cancer, lung cancer, prostate cancer, melanoma, and other malignancies [32]. The increased expression of BiP is functionally related to the prosurvival response of cancer cells to major environmental stress. This may occur through a molecular complex formation and inhibition of BIK, a proapoptotic protein [33]. BiP has been also shown to interact with and suppress the activation of caspase-7, preventing apoptosis [34]. Furthermore, BiP is positively regulated by the mitogen-activated protein kinase (MAPK) pathway. In melanoma cells, inhibiting such pathway decreases BiP expression leading to increased caspase-4 mediated ER stress induced apoptosis [35]. BiP is also responsible for cancer resistance to different anticancer therapies. Notably, BiP expression level in breast cancer may have a prognostic value [36]. BiP can therefore represent a molecular target; its inhibition may reduce its cytoprotective effects in combination with photodynamic therapy [37]. In gastric cancer cells treated with multidrug resistance cell-specific binding peptide, decreased BiP expression has been reported and this event prevents multidrug resistance [38].

Human lymphomas demonstrated significantly higher levels of UPR activation compared with normal tissues. In lymphoma models, c-Myc activates the PERK/eIF2*α*/ATF4 arm of the UPR, leading to increased cell survival via the induction of cytoprotective autophagy by PERK activation [39]. Accordingly PERK deletion inhibits mammary tumor development and reduces lung metastases [40]. PERK/eIF2*α* pathway largely contributes to the growth and survival of cancer under hypoxic stress [8]. In fact PERK is responsible for activation of many angiogenic genes [41]. Accordingly, PERK inhibition has been found to reduce tumor growth both* in vitro* and* in vivo* [42].

XBP1 increased expression and splicing have been found in hepatocellular carcinoma and breast cancer. It contributes to the adaptive response to ER stress and to survival under hypoxic conditions through positive regulation of BiP. In addition, XBP1 mutations have been described in tumor cells from patients with multiple myeloma [43–45]. XBP1 overexpression in myeloma cells has also been demonstrated and it seems to be critical for multiple myeloma induction. XBP1 therefore represents a regulator of plasma cell differentiation [46]. Interestingly, inhibition of XBP1 splicing has been shown to reduce multiple myeloma cells growth [47]. Furthermore IRE1*α* may induce XBP1 splicing thus inducing cellular proliferation through increased expression of cyclin A1, a cell cycle regulatory protein [48]. Accordingly IRE1*α* inhibition has been shown to sensitize multiple myeloma cells to ER stress and reduce their survival [49].

ATF6 is overexpressed in many human solid tumors and is involved in promoting proliferation and survival under nutrient deprivation conditions [50]. The active Ser245-phosphorylated ATF6 is overexpressed in non-small-cell lung cancer cells [51]. Remarkably ATF6 expression contributes to cancer formation by negatively regulating genes involved in cellular senescence [52]. It also mediates survival through upregulation of LC3B, a component of the autophagosomal membrane. In liver cancer ATF6 is also responsible for upregulation of XBP1 expression and the activity of both ATF6 and XBP1 increases BiP expression, leading to hepatocarcinogenesis [45].

Altogether these data explain why the inhibition of ER chaperones level or of one arm of the UPR components has been recently suggested as potential cancer therapies [53]. These approaches may inhibit UPR adaptive and prosurvival pathways leading to increased sensitivity to anticancer therapy.

Remarkably, persistent ER stress and UPR activation by pharmacological approaches can switch the cytoprotective functions of UPR into cell death programs. Therefore both repression of UPR-dependent survival signals [54] and sustained UPR induction may have beneficial and therapeutic effects against cancer. Some antitumoral agents (e.g., cannabinoids) activate ER stress as the primary mechanism to promote cancer cell death [55, 56]. It is still not known if sustained ER stress and UPR activation can induce tumor cell death activating additional unknown cell death programs. Future work needs to be done to address this issue in the context of cancer therapy.

## 4. ER-Associated Degradation (ERAD)

ER-Associated Degradation (ERAD) represents an additional cellular adaptive pathway that contributes to restoring ER homeostasis by targeting unfolded/misfolded proteins toward proteasome-mediated degradation. By this pathway such proteins are translocated from ER into the cytosol where they are polyubiquitinated and degraded by the proteasome [57]. The transport into the cytosol involves Sec61 translocation channel as well as other factors identified in ERAD yeast mutants [58, 59]. Indeed the ERAD pathway is conserved from yeast to humans and deletion of ERAD-mediating factors leads to significant UPR induction [60], thus showing significant cross talk between such two pathways. Proteins not correctly folded are firstly selected by molecular chaperones. Then ERAD substrates are modified through ubiquitin binding via specific E3 ubiquitin ligases located near ER membrane. Such modification targets proteins to the proteasome located in the cytoplasm [61].

Among the numerous molecular players in mammalian ERAD pathways, the ER-membrane resident ubiquitin ligase Hrd1 forming a complex with SEL1L [62] plays an important role.

While the role of ERAD pathway in cancer is not fully elucidated, SEL1L has been shown to be involved in cancer pathogenesis. Remarkably, SEL1L overexpression inhibits cell proliferation, growth, motility, and invasion in pancreatic cancer cells. Furthermore, correlation between SEL1L protein levels and poor prognosis has been reported in breast carcinoma patients and other cancers [63]. However further studies are needed to clarify mechanisms underlying SEL1 control of tumorigenesis.

## 5. ER Stressors in Cancer Microenvironment

**Figure 2:**
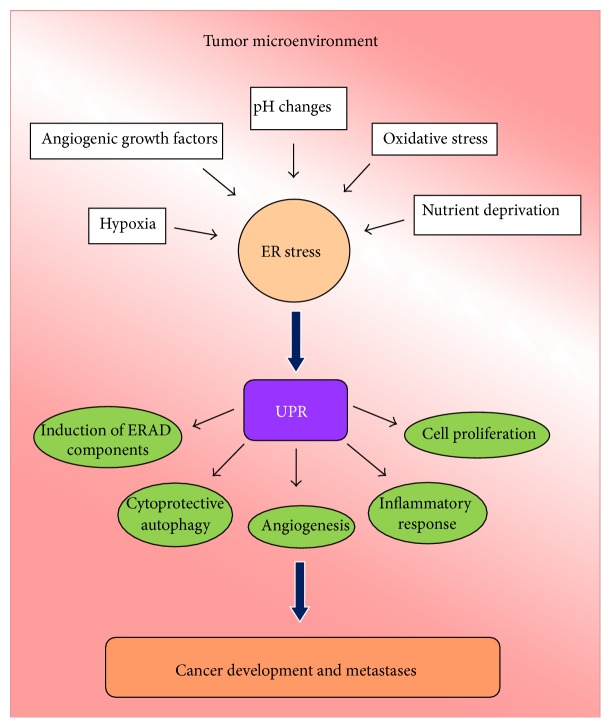
Tumor microenvironment factors activate ER stress and UPR responses leading to cancer development and metastases.

UPR activation in transformed cells is attributed to both intrinsic and extrinsic factors. Hyperactivation of oncogenes or loss-of-function mutations in tumor suppressor genes may increase protein synthesis and translocation into the ER in response to high metabolic demand and consequently UPR is activated. In addition, certain types of cancer cells are highly secretory and therefore prone to constitutive UPR activation. Defects in glycoprotein and lipid biosynthesis as a consequence of DNA mutations might also contribute to the induction of ER stress [2]. Mutations in oncogenes and tumor suppressor genes have been shown to inhibit ER stress induced apoptosis [39, 64]. In addition mutations in molecular components of the UPR pathways may also directly contribute to enhanced cancer cell survival upon stress. For example some IRE1*α* mutants, identified in human cancers, are unable to display proapoptotic RIDD function, thus showing increased cell survival [65]. Furthermore, enhanced activation of IRE1 may have a cytoprotective effect leading to cancer progression via XBP1 mRNA splicing [66]. In response to chronic stress, normal cells usually attenuate the IRE1*α*–XBP1 and ATF6*α* pathways, so that the apoptotic signals dominate. On the contrary, some cancer cells have constitutive activation of IRE1*α*–XBP1 thus inhibiting apoptosis [67, 68]. Although tumors secrete angiogenic factors to promote angiogenesis, this is often not sufficient to satisfy the elevated tumor metabolic requirements. In addition to hypoxia, cells in developing tumors are subject to glucose deprivation, lactic acidosis, oxidative stress, and decreased amino acid supplies [2]. All these changes in the microenvironment contribute to activating the UPR (see Figure 2).

### 5.1. Hypoxia

Tumor growth with defective microcirculation leads to hypoxia, which activates the UPR [69–72]. Since UPR increases cellular survival and proliferation, these events may produce a positive loop further promoting tumor growth and increasing hypoxia within the tumor. Therefore, hypoxia-mediated UPR activation appears to be essential for tumor cell survival. Although there is a general inhibition of translation under moderate-extreme hypoxia, some proteins are induced under low O_2_ conditions including HIF-1 and its downstream targets [73]. Hypoxia induces Ser51 phosphorylation of the translation initiation factor eIF2*α* via PERK activation, and this is required to downregulate protein synthesis. Hypoxia tolerance is also dependent on the upregulation of ATF4, a downstream effector of eIF2*α* phosphorylation, both* in vitro* and* in vivo*. The UPR pathway mediated by activation of IRE1 and its downstream target XBP1 is also required to counteract hypoxic conditions leading to tumor growth. Human fibrosarcoma and lung carcinoma cells upregulate BiP level and XBP1 splicing under hypoxia, whereas human colon cancer cells upregulate PERK-dependent phosphorylation of eIF2*α* and ATF4 translation [74]. It has been demonstrated that XBP1-deficient tumor cell survival is reduced under hypoxic conditions* in vitro*, and these cells are unable to develop tumors* in vivo*. Conversely, spliced XBP1 expression restores tumor growth [8]. Another potential UPR trigger in hypoxic conditions is the ER oxidase 1*α* (ERO1*α*) enzyme that catalyses disulfide bond formation in an oxygen-dependent manner. ERO1*α* activity is reduced by low O_2_ conditions, thus compromising correct protein folding and activating UPR [75]. All these hypoxic-modulated molecular responses are differently activated depending on oxygen level and hypoxia duration.

### 5.2. Oxidative Stress and Inflammatory Stress

ROS play a causal role in tumor development and progression by promoting genetic and epigenetic alterations and inducing protumorigenic signaling [76].

Under protein overload conditions, ROS are generated in the ER as a part of an oxidative folding process. ROS can target ER resident proteins, enzymes, and ER based calcium (Ca^2+^) channels, leading to calcium release from the ER into the cytosol and ER stress signaling. Increased cytosolic calcium and calcium entry in mitochondria from ER via MAM-associated channels can stimulate mitochondria metabolism to further ROS production [77]. Increased ROS accumulation may also occur as a consequence of excessive nutrients thus inducing ER stress and activating the UPR [2]. As a consequence of ROS increase, PERK is activated. PERK activation may limit oxidative DNA damage through Nrf2 transcription factor induction, thus promoting cancer cell proliferation [40].

ROS signaling cooperates with UPR pathway leading to inflammatory responses [78]. Proinflammatory stimuli (e.g., TLR ligands and cytokines) trigger ER stress further amplifying inflammatory responses. The IRE1*α*–TRAF2 complex can recruit apoptosis signal-regulating kinase 1 (ASK1) and activate JUN N-terminal kinase (JNK), increasing the expression of proinflammatory genes through enhanced AP1 activity [79].

Interestingly, all three UPR branches activate NF-*κ*B which is an important transcriptional regulator of proinflammatory pathways [80]. The PERK–eIF2*α* and ATF6*α* branches of the UPR activate NF-*κ*B through different mechanisms. PERK and eIF2*α* signaling stops protein synthesis and increases the NF-*κ*B/I*κ*B ratio, reducing I*κ*B half-life and leading to NF-*κ*B nuclear translocation [81, 82]. ATF6*α* activates NF-*κ*B via AKT phosphorylation [83, 84]. NF-*κ*B can be also activated through binding to the IRE1*α*–TNF receptor-associated factor 2 (TRAF2) complex in response to endoplasmic reticulum (ER) stress, leading to recruitment of the I*κ*B kinase (IKK), I*κ*B phosphorylation and degradation, and nuclear translocation of NF-*κ*B [85].

ER stress also induces transcription of proinflammatory cytokines in macrophages and promotes the type M2 macrophage phenotype that in turn supports tumor growth [86]. In addition ER stress, in combination with TLR agonists, by stimulating IL-23 in dendritic cells, may favour development of T helper 17 (TH17) and tumor growth [87, 88].

### 5.3. Nutrient Deprivation and Acidosis

Some other environmental factors indirectly induce ER stress and UPR activation. Amino acid deprivation activates eIF2 K4 to phosphorylate eIF2*α*. Low glucose availability affects protein glycosylation and ATP production leading to misfolded proteins accumulation within the ER [89]. Glucose shortage also leads to disturbed ER–Ca^2+^ homeostasis that is mediated by reduced sarcoplasmic/endoplasmic reticulum calcium ATPase (SERCA) activity. At low glucose concentration SERCA pump inhibition leads to PERK activation [90]. BiP is also upregulated at low glucose concentration. Interestingly BiP identification was originally made in low glucose experiments [91]. Also XBP1 is involved in response to glucose deprivation. In particular in the XBP1s reporter mouse model, which develops spontaneous mammary tumors, XBP1 splicing was found to increase upon exposure to a nonmetabolizable glucose analog that simulates glucose deprivation [92]. Tumor cells adapt to low glucose levels by switching to a high rate of aerobic glycolysis, which is known as the Warburg effect [93, 94]. The consequent lactic acid production reduces the pH, and low pH is an important feature of the tumor microenvironment, promoting tumor survival and progression also* via* UPR by regulating several BCL-2 family members and CHOP [95].

### 5.4. Angiogenic Growth Factors

Growth factors synthesized and released within the tumor microenvironment may contribute to UPR activation. In cancer cells a direct link of UPR with growth factors is still to be investigated in detail; however different forms of PDGF (i.e., both PDGF-B and PDGF-A) are known to induce ER stress in nontumor models such as a vascular injury model [96], transgenic mouse crystalline lens models [97] and renal fibrosis [98]. Such data may suggest that the observed role of PDGF family members in melanoma and angiogenesis [99, 100] and in different tumors [101–103] may relate, at least to some extent, to ER stress inducing properties. As far as FGF family members are concerned, one recently published study [104] demonstrates that FGF-2 prevents ER stress induced cancer cell apoptosis in a Nck 1 (Src homology 2/3 domain-containing protein) mediated way. Further, Wang and colleagues recently demonstrated that glucose deprivation induces a PERK/ATF4-mediated UPR which leads to a proangiogenic action by stimulating the expression of a number of proangiogenic factors such as VEGF and FGF-2 and inhibiting the expression of antiangiogenic factors such as THBS1, CXCL14, and CXCL10 [105]. Moreover, VEGF has been shown to induce UPR in an ER stress independent manner, via PLC*γ* and mTORC1, indicating these players as constitutive parts of the VEGF signaling machine [106]. On the other hand, UPR has been shown to prevent inositol-requiring protein 1 (IRE1) *α* and ATF6-mediated VEGF degradation [107]. Finally, UPR, via IRE1*α*/XBP-1, PERK-ATF4, and ATF6*α* pathways, acts as an upstream regulator of VEGF transcription, directly affecting angiogenesis [108].

## 6. Recent Evidences on the Direct Role of UPR Regulation in Counteracting Prostate Cancer and Melanoma

Cancer cells generally display increased apoptosis resistance as compared to normal cells, thus bypassing ER stress-induced cell death [109]. Current strategies to counteract cancer growth aim at exacerbating ER stress thus stimulating prodeath UPR. Recent studies underlie the UPR targeting in different cancers such as prostate cancer [110] or melanoma [111]. While UPR role in tumors such as breast cancer [112] and lung cancer [113] has been reviewed in the last three years, recent reviews focused on “melanoma and UPR” or “prostate cancer and UPR” are lacking.

The key UPR relevance in response to cancer may be underlined by the observation that UPR activators may represent valuable novel therapeutic targets in a number of cancer conditions. For instance, SMIP004, a potent inhibitor of prostate and breast cancers growth, has been shown to achieve its proapoptotic effect by altering mitochondrial respiration and activating a MAPK-dependent proapoptotic effect downstream UPR [110]. In addition, death of prostate cancer cells such as PC3 and PNT1a has been observed upon treatment with a standardized green tea extract, acting via UPR activation, leading to cell cycle arrest at G2/M checkpoint in PC3 cells and at G0/G1 checkpoint in PNT1a cells [114]. Furthermore, subtilase cytotoxin catalytic subunit has been shown to sensitize prostate as well as lung cancer cells to photodynamic therapeutic treatments, mostly inducing cell death rather than apoptosis. This effect is related to subtilase targeting of GRP78, a major player in UPR regulation [37]. GRP78 appears to be a potentially relevant molecular target; in fact, also in melanoma, targeting GRP78 via subtilase has been shown to be an effective way to increase the proapoptotic effect of drugs such as fenretinide or bortezomib [115, 116]. Finally, combination of Pim kinase inhibitor and BCL2-antagonist has been shown to induce a strong* in vitro* and* in vivo* apoptosis in prostate cancer cells, mediated by Noxa protein activating UPR [117]. Interestingly, a Noxa-dependent proapoptotic effect has been also observed in melanoma cells, induced by another UPR activator named aurin [118].

## 7. Concluding Remarks

The UPR appears to adjust cancer microenvironment and represents a mechanism underlying resistance against cancer therapy [119]. Transformed cells may exploit UPR as a survival strategy to survive in a stressful microenvironment. While most studies demonstrate crucial roles for UPR signaling in tumor growth and chemoresistance, only recently UPR activation has been demonstrated to occur during oncogenic transformation and cancer development since UPR signaling molecules have been shown to interact with oncogenes and tumor suppressor genes. Further studies are necessary to understand in more detail the exact interaction of the involved signaling pathways [2]. Identification of such key players has the potential to select additional novel therapeutic approaches to improve the antitumor treatments. Further, selective inhibitors of the ER stress response may be revealed to be useful to counteract drug resistance [89, 120].

Recently several IRE1*α* inhibitors, namely, STF-083010, 3-ethoxy-5,6-dibromosalicylaldehyde, 2-hydroxy-1-naphthaldehyde, toyocamycin, and irestatin, have been found to induce apoptosis in pancreatic cancer cells [121] and in malignant myeloma cells [122]. Such IRE1*α* inhibitors have shown promising* in vitro* effects, in combination with other drugs [121]. IRE1*α* inhibitors clinical potential also comes from the observation that IRE1*α* inhibition sensitizes cancer cells to apoptosis induced by oncolytic virus therapy [123]. As an example, the novel therapeutic agent eeyarestatin I targets p97, an ATPase involved in the transport of ubiquitinated proteins, and blocks ERAD pathway inducing cancer cell death [124].
